# The Relative Role of Semantic and Sublexical Processes in Reading, Writing and Repetition: Evidence from a Follow-Up Study

**DOI:** 10.3233/BEN-2009-0255

**Published:** 2010-06-10

**Authors:** Flavia Mattioli

**Affiliations:** Neuropsychological UnitSpedali CiviliBresciaItaly

**Keywords:** Aphasia, summation hypothesis, surface dyslexia

## Abstract

The evolution in time of a number of language tasks in a longitudinal study of a 61-year-old aphasic patient is described. The patient, examined twice, in a 10 month follow-up, showed a dissociation between preserved reading with respect to impaired other modalities as well as a qualitative change in errors' type. A reduction of neologisms and phonologically based errors, with a concurrent increase of semantic paraphasias in naming and repetition, as well as an amelioration in reading, with a reduction of stress assignment errors was exhibited at the follow-up. The results are interpreted by postulating an improved performance of the phonological output processes, allowing non-phonologically based errors to emerge, thus revealing the underlying semantic damage. The Summation Hypothesis [14] seems a general framework better interpreting these findings, more than highly specialized production models, which could explain separately only different modalities’ impairments.

## 1. Introduction

The cognitive procedures for deriving sound from print during reading are supposed to be at least two [9, 10,20].

One, generally referred to as a *lexical process* involves relating word in the orthography to similar units in the phonological lexicon. According to a *direct acces* view, the lexical procedure involves first retrieving meaning from orthography and then using the words' semantics to address its phonology. The second procedure is assumed to use knowledge in the regularities of relationships between ortography and phonology to convert the representation of the stimulus into a phonologically plausible sequence of phonemes and is referred to as the non lexical or *sublexical process* (the orthography to phonology conversion OPC or grapheme to phoneme conversion GPC system, which allows to convert a written cluster of vowels and consonants in the corresponding spoken sound independently from the lexicon).

This mechanism allows for the ability to read aloud unknown words and sequences of letters not corresponding to real words. Given this theory, if brain damage impairs the lexical process, but spares the non-lexical (rule-based) method of reading, the patient will be able to read nonwords successfully, but will make regularization errors with exception words, that is to say, the patient will exhibit surface dyslexia [26]. Surface dyslexia has been frequently described in English speakers [8,22], whereas it has been seldom reported in Italian native speakers. This because unlike English, which contains a relevant number of words that have exceptional print-to-sound mappings (opaque orthography), Italian is considered a "transparent" language.

However, in Italian stress assignment is largely unpredictable. In several Italian words, the stress assignment's exact location can be determined only by means of lexical route reading (Italian would

ISSN 0953-4180/09,10/$17.50 © 2009/2010 – IOS Press and the authors. All rights reserved be considered “segmentally” transparent and “suprasegmentally” opaque). For instance, only the lexical knowledge allows to correctly attribute the stress of words such as “fràgola” (*strawberry*), because in Italian the most frequent pattern of stress assignment is the penultimate syllable (84%) and only 12% of words are stressed on the antepenultimate syllable [32].

The *dual-route* model poses that an expert reader normally uses the lexical route and uses the non-lexical or *sublexical* one for unknown words or non-word reading [9]. Furthermore, for highly regular or transparent languages (i.e. a language whose strict relationship between graphemes and phonemes allows the great majority of words to be read correctly by way of GPC rules), such as Spanish, Italian, Serbo-Croatian and Korean, it has been suggested that readers develop more sublexical procedure than for opaque languages (English, Chinese), which use mainly the lexical procedure.

A third route, a *non semantic lexical route*, has also been posited, based on connections from a word’s orthography to its phonology that bypasses semantics. This could account for good reading of irregular words in the face of damaged lexical route, as shown by their lack of comprehension of the same words [28].

Hillis and Caramazza (1991, 1995) described individuals whose clinical pattern might be accounted for without positing a third route. For example JJ made semantic errors in naming and comprehension, in spite of correct reading of the same items, as well as of non words. The authors proposed that an integration across lexical and sublexical processes would be able to account for such a pattern, instead of postulating a third route.

In the *Summation Hypothesis*, they assumed that lexical representations are activated and selected in the phonological and orthographic output lexicons by the joint operation of both the lexical-semantic system and the sub-lexical mechanisms.

According to this theory and differently from the dual route model, the sub-lexical mechanism is supposed to have an important role also in reading of known words, since lexical representation is selected for output by summing together information from both the semantic and the sub-lexical GPC system.

For instance, when reading “gatto“ (*cat*), both the lexical-semantic and the GPC system would be activated in parallel. Due to the function of the lexical-semantic system, the phonological form is activated by the semantic information, which also activates, though to a lesser degree, other semantically related entries such as, “leone” (*lion*), “cane” (*dog*), and so forth. Concurrently, the conversion procedure transforms the orthographic sequence in one or more phonologically plausible sequences. Thus, the activation received by the conceptual information is enhanced by the sub-lexical phonological information assembled by the sub-lexical mechanisms of conversion.

In general, this theory predicts that reading of known words depends not only upon the operation of the lexical-semantic system, but also upon the operation of the sub-lexical GPC system.

It would also explains various other performance patterns, i.e. high rates of semantic errors in picture naming, but not in oral reading.

The italian aphasic patient ECA [24] produced semantic errors in all lexical production and comprehension tasks, except reading aloud, because even though he was damaged in the semantic component, his substantial sparing of sublexical GPC effectively blocked the production of such errors in reading.

Under normal circumstances, the activation of lexical representations from the semantic component is sufficient to determine the correct output, but if the activation deriving from the semantic system is insufficient to select the correct output, the activation deriving form the sublexical GPC procedure will help to produce the correct response.

Consistent with the *Summation Hypothesis* is also the reported dissociation in patient SA, who was diagnosed to be affected by semantic dementia [33] whose poor performance in object naming and in semantic memory tasks contrasted with his moderate difficulties in reading and spelling. SA was able to read many irregular words, which he could not name on visual presentation because he used a partial semantic description to constrain the responses deriving from the unimpaired GPC.

The possible role of the *Summation Hypothesis* in the explanation of the spontaneous recovery of aphasia is not known and follow up studies of aphasic patients in this view are scanty. It is possible that a different quantitative damage to both the lexical and the sublexical procedures may determine and explain a different error pattern in the aphasic patient.

In this paper, we present a follow-up study of an Italian-speaking aphasic patient, whose relevant characteristic is the preservation of reading with respect to the other language output modalities, during the whole follow up.

In particular, he could correctly read several irregular words, in spite of his severe damage to other language output tasks. The evolution of such impairments is worth noting and could appropriately be interpreted in the framework of the *Summation Hypothesis*.

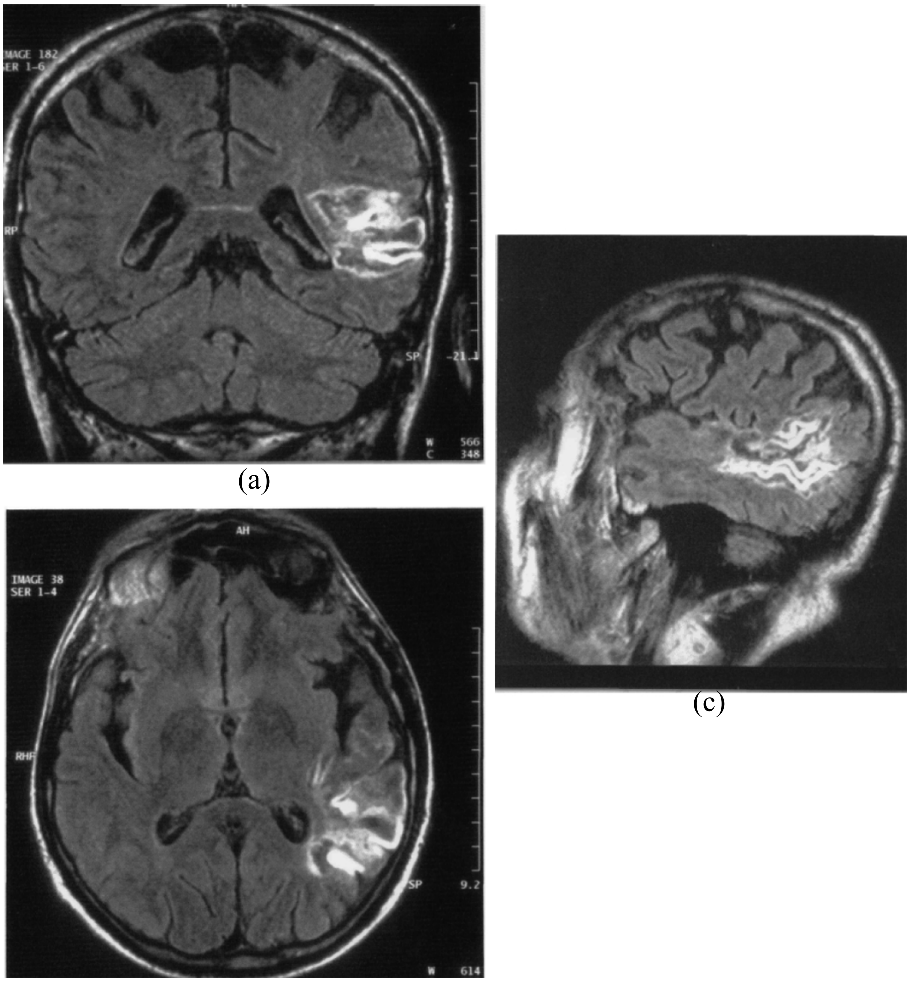

(a) (b) (c)

Fig. 1. Legend: MRI (performed 15 days post onset) sagittal, lateral and assial images showing the lesion extension in left temporal and parietal cortices.

## 2. Case description

MS was a 61-year-old, right-handed Italian male with five years of education. He was a labourer before retiring and his significant medical history includes: insulin-dependent diabetes, dyslipidemia and smoking. In January 2001, he was hospitalized because he developed sudden global aphasia. The patient was not able to comprehend even simple orders, as well as single words of common objects between two distractors. His spontaneous speech was paraphasic and consisted of incomprehensible utterances. Neurological examination showed right homonymous hemianopia; neither hemiparesis, nor hemisensory loss were detected.

The CT scan performed the day after the stroke showed a left temporal ischemia. Brain MRI (Fig. 1) showed a lesion in the left hemisphere involving the superior temporal gyrus, the supramarginal gyrus, and the posterior parietal cortex (Brodman's areas 5 and 7) due to an ischemic stroke in the area of the left middle cerebral artery.

The patient was referred to our Neuropsychological Unit in May 2001 for neuropsychological testing. He was evaluated twice: once in May and once in October 2001.

The first neuropsychological evaluation revealed normal intellectual abilities (Raven's Coloured Progressive Matrices 28.5/36; normal age, education, sex matched controls; cut off 17.5) [27]. A mild buccofacial apraxia, but neither limb nor constructional apraxia, was observed.

Table 1
MS's AAT performance

| AAT | Range | Score | RP* | PT** | Severity |
|---|---|---|---|---|---|
| Token Test (errors) | 50–0 | np | | | Severe |
| Repetition | 0–150 | 0 | 0 | 22 | Severe |
| Written language | 0–90 | 22 | 26 | 44 | Medium |
| Naming | 0–120 | 20 | 15 | 39 | Severe |
| Comprehension | 0–120 | 78 | 37 | 47 | Medium |

Legend: *percentile rank, **T score.
Np: not performable.

Table 2
MS'S BADA performance (errors' percentage)

| | 1st evaluation | | 2nd evaluation | |
|---|---|---|---|---|
| Test | Errors | % | Errors | % |
| Phon. auditory discrimination | np | | 12/60 | 20 |
| Phon. aud./vis discrimination | 13/60 | 22 | 10/60 | 17 |
| Auditory lexical decision | np | — | 22/80 | 27 |
| Visual lexical decision | 29/80 | 36 | 15/80 | 19 |
| Single Words | | | | |
| – repetition | 45/45 | 100 | 45/45 | 100 |
| – dictation | 46/46 | 100 | 46/46 | 100 |
| – reading | 12/92 | 13 | 3/92 | 3 |
| – delayed copy | 3/10 | 30 | 1/10 | 10 |
| Non Words | | | | |
| – repetition | 36/36 | 100 | 31/36 | 86 |
| – dictation | 24/24 | 100 | 24/24 | 100 |
| – reading | 5/45 | 11 | 1/45 | 2 |
| – delayed copy | 1/6 | 17 | 1/6 | 17 |
| Oral Naming | 28/30 | 93 | 26/30 | 87 |
| Written naming | np | — | 20/22 | 91 |
| Auditory comprehension nouns | 2/40 | 5 | 6/40 | 15 |
| Visual comprehension nouns | 0/40 | 0 | 0/40 | 0 |
| Auditory comprehension verbs | 4/20 | 20 | | |
| Visual comprehension verbs | 1/20 | 5 | | |

In order to investigate non-verbal semantic information, MS was tested on the three-picture shortened version of the Howard & Patterson's [7] Pyramids and Palm Trees and his score was 27/30 correct (mean age and sex matched controls' performance: 29.3/30) indicating a mild semantic knowledge impairment.

Performance on the auditory version of the Peabody Picture Vocabulary Test [12] was 94/150 correct.

He was submitted to an extensive language examination by means of the Italian version of the Aachener aphasia test (AAT; Luzzati et al., 1991) which revealed a Wernicke aphasia profile (see Table 1) and the Battery for the analysis of aphasic deficits [25] (see Table 2).

Overall, his AAT performance appeared to be severely impaired. MS's comprehension score was better for single words (25/30 and 24/30 correct for both oral and written comprehension respectively, the correct target has to be indicated between 1 phonological or semantic distractor and 2 non related alternatives) than for sentences (14/30 for oral and 15/30 for written comprehension; the correct target has to be indicated between 3 morphologically/ grammatically similar alternatives).

The single word analysis (BADA) results showed multiple levels of impairment.

Deficits were found in the sublexical tasks: phonological auditory discrimination (i.e. TA-PA equality auditory decision task) could not be performed and in phonological auditory to visual discrimination (i.e. TA usually matched with PA for equality judgment) MS made 13/60 errors.

In the lexical decision task (i.e. decision if a visually presented word/nonword is a real word) MS scored 29/80 errors on visual decision, whereas auditory decision (i.e. decision if an auditory presented word/nonword is a real word) could not be performed.

Both repetition and dictation were impaired. A complete impairment was evident for repetition with 45/45 errors in word repetition and 36/36 errors in nonword repetition. The same impairment was evident for nonword dictation, with 24/24 errors and for word dictation with 46/46 errors. In the delayed copy task he scored 1/6 errors for non-words and 3/10 for words. Oral and written naming were also impaired (28/30 errors and not performable, respectively).

Unexpectedly, MS's oral reading of single words was only mildly impaired (he made 12/92 errors for words and 5/45 errors for non-words).

In single word auditory comprehension (i.e. indicating the correct figure corresponding to an auditory presented word/verb between the target and the phonological or semantic alternative) his performance was 2/40 errors for nouns and 4/20 errors for verbs. Visual comprehension was performed somewhat better, with 0/40 errors for nouns and 1/20 errors for verbs.

In all the production tasks, except reading, errors were mainly neologisms and stereotipypes whereas reading errors were phonemic paraphasias. No semantic errors were found.

Summing up, these observations allow us to conclude that MS was severely impaired in repetition and dictation, both with words and nonwords, as well as in object naming (written and oral), in auditory lexical decision and phonological auditory discrimination. On the other hand, reading aloud was the better-preserved modality.

This dissociation in the output modalities was further investigated in both examinations.

## 3. Experimental study of language

In order to better investigate the dissociation observed in MS's language, i.e. a relative sparing of reading aloud with regard to the other output modalities, a list of 72 picturable words was presented in the following modalities: naming, dictation, repetition, auditory and visual comprehension, and reading. This list consisted of two sets, respectively, containing trisyllabic and tetrasyllabic words, each divided in two sub lists with the stress on the penultimate and on the antepenultimate syllable.

Auditory and visual comprehension tasks consisted of pointing to either an auditory or visually presented figure within two distractors, one semantically related and one unrelated. Because of the target word's length, we deliberately omitted a phonological distractor, since it was not possible to find it for every item.

The sub lists were comparable for CV structure, frequency and familiarity. The criteria for word inclusion were:

– Phonological structure (sequence consonant- vowel)
– Word frequency (words with high frequency in common Italian language, considering the subject's education), excluding words included in highly specific semantic categories (for example technical terminology of particular work areas)
– Word length. Words of three to four syllables were chosen with a balanced CV structure
– Word imageability. All words were highly imageable, belonging to the semantic categories of animals, body parts, foods, clothes, toys and tools, with typical representation.
– Stress assignment. As explained before, the sub lists of the trisyllabic and tetrasyllabic words were divided in order to construct four groups. The first group included 19 trisyllabic words stressed on the penultimate syllable (e.g. caròta, *carrot*), the second consisted of 17 trisyllabic words stressed on the antepenultimate syllable (e.g. tàvolo, *table*), the third included 20 tetra syllabic words stressed on the penultimate syllable (e.g. lampadàrio, *chandelier*) and finally the fourth group included 16 tetrasyllabic words stressed on the antepenultimate syllable (e.g. semàforo, *traffic light*).

In order to evaluate the patient's errors in the different output modalities, we used the following criteria (a variation of the criteria indicated in [21]):

Correct (C): Phonologically or orthographically accurate production of the target name in the production trials and pointing to the correct item in the comprehension tests (e.g. giornale-*newspaper*: "giornale")

Phonological paraphasia (P): non-lexical error which is phonologically related to the target, without transpositions and with few substitutions, no more than 1/3 of the word's letters (e.g. forbici-*scissors*: "barbici")

Correlated neologism (Cn): non-lexical error which is related phonologically to the target but with transpositions and many substitutions (e.g. anatra-*duck:*"atrana")

Neologism (N): a non-lexical error where no evident phonological relationship to the target is apparent (e.g. semaforo-*traffic light*: " trincon")

Phonological paraphasia on a semantic paraphasia (PS): non-lexical error which is phonologically related to a semantic paraphasia (e.g. peperone-*pepper*: "pemodori- *tematoes*). In this case the patient made clear indication of the intended semantic item.

Semantic paraphasia (S): any error which bears a semantic but no phonological resemblance to the target (e.g. manica- *sleeve*: "giubbino"- *jacket*)

Circumlocution with a semantic paraphasia (C+S): a informative description with a word semantically related to the target (e.g. lavatrice-*washing machine:*"per pulire l'acqua"- *it is to clean the water*)

Circumlocution (C): a semantically informative description of the target without reporting the name (e.g. calendario-*calendar:*"dove puoi leggere il giorno dell'anno"-*where you can read the day of the year*)

Stress assignment error (St): a correct response with a wrong stress assignment (e.g. micròfono-*microphone*: "microfòno")

Unrelated word (UW): a lexical error which is neither phonologically nor semantically related to target (e.g. margherita-*daisy:*"olio"-*oil*)

Omission (O): the patient does not produce an answer, but he says, for example,

"non so"-*I don't know*, or "è troppo lunga, non riesco."-*it's too long, I can't . . .*

No formal rehabilitation was performed because the family could not provide any help with transportation.

## 4. Results

The main result of single word processing in the 72-word test at the first examination (Table 3) is the relative sparing of reading (62.5% errors) with respect to the other output modalities (98 to 100% errors). McNemar test results confirm the superiority of reading with respect to the other output modalities, either on the first evaluation or on the follow-up (in every comparison $p < 0.01$).

The previously reported error classification reveals that MS's score is 72/72 (100%) errors in both repeti-

Table 3

Error's type in 72 words naming (nam.), repetition (rep.), dictation (dict.), reading (read.) and comprehension (aud. and vis. comp) in the first and in the second evaluation

| Error'type | O | O | N | N | CN | CN | UW | UW | P | P | C | C | S | S | PS | PS | C+S | C+S | St | St | TE | TE | TE % | TE % |
|---|---|---|---|---|---|---|---|---|---|---|---|---|---|---|---|---|---|---|---|---|---|---|---|---|
| Evaluation | 1st | 2nd | 1st | 2nd | 1st | 2nd | 1st | 2nd | 1st | 2nd | 1st | 2nd | 1st | 2nd | 1st | 2nd | 1st | 2nd | 1st | 2nd | 1st | 2nd | 1st | 2nd |
| Nam. | 0 | 5 | 55 | 19 | 2 | 3 | 3 | 6 | 5 | 5 | 2 | 13 | 4 | 7 | 0 | 2 | 0 | 2 | 0 | 0 | 71 | 62 | 99 | 86 |
| Rep. | 0 | 2 | 71 | 41 | 1 | 6 | 0 | 2 | 0 | 7 | 0 | 2 | 0 | 7 | 0 | 0 | 0 | 1 | 0 | 0 | 72 | 68 | 100 | 94 |
| Dict. | 0 | 0 | 70 | 68 | 2 | 3 | 0 | 0 | 0 | 1 | 0 | 0 | 0 | 0 | 0 | 0 | 0 | 0 | 0 | 0 | 72 | 72 | 100 | 100 |
| Read. | 0 | 0 | 0 | 0 | 2 | 0 | 0 | 0 | 20 | 1 | 0 | 0 | 0 | 0 | 0 | 0 | 0 | 0 | 23 | 11 | 45 | 12 | 62.5 | 17 |
| aud comp | 0 | 0 | 0 | 0 | 0 | 0 | 1 | 0 | 0 | 0 | 0 | 0 | 7 | 3 | 0 | 0 | 0 | 0 | 0 | 0 | 8 | 3 | 11 | 4 |
| vis. comp. | 0 | 0 | 0 | 0 | 0 | 0 | 0 | 0 | 0 | 0 | 0 | 0 | 1 | 1 | 0 | 0 | 0 | 0 | 0 | 0 | 1 | 1 | 1 | 1 |

**Legends**

O: omissions; N: neologism; CN: correlated neologism; UW: unrelated word; P: phonological paraphasia; C: circumlocution; S: semantic paraphasia; PS: phonological paraphasia on a semantic paraphasia; C+S: circumlocution with a semantic paraphasia; St: stress assignment error; TE: total errors.

Table 4
Stress assignment errors in trisyllabic and tetrasyllabic words

| N° syllables | penultimate sillable stress 1° evaluation | antepenultimate sillable stress 1° evaluation | penultimate sillable stress 2° evaluation | Antepenultimate sillable stress 2° evalutation |
|---|---|---|---|---|
| trisyllabic | 0/19 | 14/17 | 1/19 | 4/17 |
| tetrasyllabic | 0/20 | 9/16 | 0/20 | 6/16 |

tion and dictation (71 neologisms and 1 correlated neologism in repetition, 70 neologisms and 2 correlated neologisms in dictation).

Naming performance (99% errors) consists mainly of phonologically based errors.

In auditory and visual comprehension tasks, MS makes less errors (11% and 1% errors respectively). The errors are mostly semantic.

This better performance in comprehension tasks may be ascribed, in our opinion, both to a much lower impairment in the comprehension modalities and by the fact that the comprehension tests are comparatively easier than the expression tests.

Again, reading aloud of single words seems to be the single preserved production task: in fact, when MS reads 45/72 words incorrectly, the errors are mainly stress assignment errors (23/72).

Out of 23 stress assignment errors, 100% are on the trisyllabic and tetrasyllabic words stressed on the antepenultimate syllable (14/17 and 9/16 respectively) (Table 4). In all these items MS regularizes the position of the stress, transposing it on the penultimate syllable.

In October, 10 months after the stroke, the follow-up evaluation was performed.

BADA (Table 2) showed an improvement in all single tests except repetition and dictation.

Results of the 72-word test show a reduction and a qualitative modification of the errors (Table 3). In naming his score improves changing from 1 to 10/72 correct (Mc Nemar test; $X^2 = 7{,}11$; $p < 0.01$), in repetition from 0 to 4/72 (p. ns), in dictation the score remains unchanged and in reading it changes from 27 to 60/72 ($X^2 = 31{,}03$; $p < 0.01$).

Scrutiny error pattern (Table 3) reveals a greater number of semantic errors, compared to the first evaluation. Naming errors are, in fact, to a lesser extent phonologically based and in a greater amount semantically based (7 semantic paraphasias, 2 circumlocutions with semantic paraphasias, 13 circumlocutions, 6 unrelated words and 5 omissions), or phonologically based on semantically based. For example a phonological paraphasia complicating a semantic paraphasia, i.e. naming *melena* (similar to *mela* (*apple*) instead of arancia (*orange*) and *pemodori* (similar to *pomodori: tomatoes*) instead of peperoni (*pepper*). In repetition, a reduction of neologisms, an increase of correlated neologisms and the appearance of phonological paraphasias, semantic paraphasias, circumlocution with semantic paraphasia, circumlocutions, unrelated words and omissions were found.

Dictation remains impaired with 100% errors.

Reading significantly improves: phonological errors decrease (from 11 to 1) as well as stress assignment errors (from 23 to 11). These are respectively 4 in trisyllabic words stressed on the antepenultimate syllable, 1 in trisyllabic words stressed on the penultimate syllable and 6 in tetrasyllabic words stressed on the antepenultimate syllable. Again MS regularizes the stress position for the 10 words stressed on the antepenultimate syllable and for the word stressed on the penultimate syllable he anticipates the stress (i.e. *càndela* instead of candèla) (Table 4).

MS correctly attributes stress to 21/31 words stressed on the antepenultimate syllable (binomial distribution test $p < 0.05$).

Auditory comprehension also improves, although non significantly ($p$ = ns), changing from 64/72 to 69/72 correct, whereas visual comprehension remains 71/72 correct (1 semantic error).

In summary, while in the first evaluation phonologically based errors were prevalent in all the output modalities, upon the second evaluation, a reduction of phonologically based errors and an increase of semantic errors, circumlocution and unrelated words is observed.

Moreover, in the follow up, the patient correctly reads the irregular words in a higher number than chance, doing regularization errors as well.

## 5. Discussion

The main points of interest of this single case longitudinal study are two: first the dissociation between preserved reading and other impaired modalities and second the presence of stress assignment errors, both longitudinally observed in an aphasic patient's follow-up.

The standard language examination of MS evidences in both exams a preserved reading aloud of both words and nonwords, in contrast with a poor performance in all other output modalities.

Looking at error quality in naming and in repetition, the high number of neologisms followed by correlated neologisms and phonological paraphasias in the first evaluation suggests a greater damage to phonology than to semantic system. The follow-up shows a reduction of phonologically based errors, an increase of semantic errors, circumlocutions and unrelated words and the appearance of omissions, suggesting that the reduction in the phonological damage may allow the semantic system impairment to become more evident.

The Summation Hypothesis provides an explanation as to why reading performance should be better than in other tasks, despite the presence of deficits which appear to affect all output modalities, an explanation which is not provided by the dual route model (which is only a reading model) [10] and also the spontaneous evolution of this case could, in our opinion, be also better explained in this general framework. The *Summation Hypothesis* [14] states that, if the activation deriving from the semantic component were to be insufficient to determine the correct output, the activation deriving from the sublexical OPC procedure could help to produce the correct response, in the reading task. Moreover semantic errors in other tasks, such as word-picture matching or picture naming, would also be explained if the semantic activation procedures are underfunctioning. In fact in these tasks the information is entirely derived from the semantic system. The change in damage's degree and quality to both the semantically and sublexically based information would determine the error pattern in aphasia over time. In particular, in MS's follow up, a reduction of the damage at the phonologically based level of word processing, can be hypothesized (being the neologisms at the first evaluation substituted by correlated neologisms and phonological paraphasias in all the modalities). This would be coexisting with, an at least partially-functioning semantically based processing (allowing an increase in semantic over phonological paraphasias).

Patients' description of dissociated performances in transcoding modalities of reading and writing to dictation, with similar number of semantic paraphasias in naming tasks, have been described [1] and reported to be dependent on the availability of the GPC and PGC procedures. This study of our patient, MS is to our knowledge, the first case of a single patient's follow-up demonstrating how lexical and conversion procedures may be differently damaged and may spontaneously recover to different extents, causing a rather predictable type of aphasic errors pattern in output modalities.

A similar interpretation may be argued with respect to stress assignment errors in MS reading, which reduced from the first to the second evaluation.

Summation Hypothesis can well explain the observed reduction in stress assignment errors. Such errors' existence, proof the fact that MS pronounces words by the application of sublexical OPC procedures. Their amelioration over time, allows a better print to sound matching and can cause a predictable amelioration of reading with the expected stress "regularity" effect: i.e. the shift of the stress from the correct irregular (antepenultimate) to a regular (penultimate) position. Although in a consistent number of words, MS correctly attributes the stress also to irregular words (i.e. *làmpada*), using semantically derived informations.

Other cases of patients showing errors in stress assignment have previously been described both in cerebrovascular accidents [2,23,24] ([the last case with stress assignment errors in reading, naming and spontaneous speech]) and in degenerative diseases [5].

The recovery of such ability in MS may be interpreted as a result of the interaction of recovered phonological information with recovered semantic information in the output lexicon, although errors are still present due to its incomplete function. The availability of at least partially preserved semantic information integrated by the phonological information (by GPC procedure) has, in our opinion, allowed the correct pronunciation of the items, despite the fact that neither route may be able to select the correct entry by itself.

However, the retrieval of a stored stress pattern is not the only process used in irregular words stress assignment, but the computation of stress pattern is performed also on the basis of statistical knowledge. This results from Laganaro, Vacheresse & Frauenfelder [17], whose patient produced regular (77.5%) as well as irregular (22.2%) stress patterns in a nonword reading task.

These data, however, cannot explain the performance of our patient. Considering the 72 words (except 1 phonological paraphasia) MS reads 50 words (70%) applying the rule and 21 (30%) anticipating the stress position; a higher number than the percentage of the irregular words in the Italian language. It may be argued that in MS, a partially-preserved semantic knowledge of irregular words plays a major role in correct stress assignment.

Another stress assignment modality for non-words (26.6%) was described for normal subjects by Colombo [7]. In Colombo's experiment, nonwords were formed by adding three or four letters to the ending of real words. Her results showed a correlation between the proportion of irregularly stressed pronunciations of nonwords and the proportion of real words sharing the same ending. Colombo suggests that the speaker can compute a stress pattern on the basis of implicit phonological knowledge, which depends essentially on the segmental composition of the word's last two syllables.

In MS, an experiment with nonwords has not been studied, although data point to a major role in lexical knowledge in determining an improved stress assignment.

There have been other cases of surface dyslexic patients [6,18] who were able to read correctly low-frequency irregular words in spite of a severe damage to semantic route. Actually, they had very little comprehension of the irregular words that they can read, since there is only partial semantic support. To account for this non-lexical reading, authors postulates the "third lexical route" [11,13]. This hypothesis has been criticised by others [15,30,31]. In MS, a third lexical route for stress assignment seems inplausible, given his number of regularization errors.

In conclusion the summation hypothesis seems to be the best framework allowing the explanation of the errors pattern evolution of all the different tasks in MS.

This case represents, to our knowledge, the first follow-up study in spontaneous recovery of aphasia focussing on the interpretation of the relative roles of the lexical and sublexical procedures in reading, naming, writing and repetition. Other cases with similar error patterns and follow-up are needed in order to confirm our hypothesis.
